# Structural insights into target DNA recognition by R2R3-MYB transcription factors

**DOI:** 10.1093/nar/gkz1081

**Published:** 2019-11-16

**Authors:** Baihui Wang, Qiang Luo, Yingping Li, Liufan Yin, Nana Zhou, Xiangnan Li, Jianhua Gan, Aiwu Dong

**Affiliations:** 1 State Key Laboratory of Genetic Engineering, Collaborative Innovation Center for Genetics and Development, International Associated Laboratory of CNRS-Fudan-HUNAU on Plant Epigenome Research, Department of Biochemistry, Institute of Plant Biology, School of Life Sciences, Fudan University, Shanghai 200438, China; 2 State Key Laboratory of Genetic Engineering and Ministry of Education Key Laboratory of Contemporary Anthropology, Collaborative Innovation Center for Genetics and Development, School of Life Sciences; Institutes of Biomedical Sciences of Shanghai Medical College, Fudan University, Shanghai, China; 3 State Key Laboratory of Genetic Engineering, Collaborative Innovation Center for Genetics and Development, Shanghai Public Health Clinical Center, School of Life Sciences, Fudan University, Shanghai 200438, China

## Abstract

As the largest group of MYB family transcription factors, R2R3-MYB proteins play essential roles during plant growth and development. However, the structural basis underlying how R2R3-MYBs recognize the target DNA remains elusive. Here, we report the crystal structure of Arabidopsis WEREWOLF (WER), an R2R3-MYB protein, in complex with its target DNA. Structural analysis showed that the third α-helices in both the R2 and R3 repeats of WER fit in the major groove of the DNA, specifically recognizing the DNA motif 5′-AACNGC-3′. In combination with mutagenesis, *in vitro* binding and *in vivo* luciferase assays, we showed that K55, N106, K109 and N110 are critical for the function of WER. Although L59 of WER is not involved in DNA binding in the structure, ITC analysis suggested that L59 plays an important role in sensing DNA methylation at the fifth position of cytosine (5mC). Like 5mC, methylation at the sixth position of adenine (6mA) in the AAC element also inhibits the interaction between WER and its target DNA. Our study not only unravels the molecular basis of how WER recognizes its target DNA, but also suggests that 5mC and 6mA modifications may block the interaction between R2R3-MYB transcription factors and their target genes.

## INTRODUCTION

Transcription factors control many essential biological processes by regulating the expression of genes. According to the characteristics of their DNA-binding domains, transcription factors can be divided into different families (1), among which MYB domain-containing transcription factors constitute a large family and perform diverse functions in eukaryotes (2). MYB family transcription factors share a conserved domain architecture, with a DNA-binding domain (MYB domain) consisting of 1–4 imperfect repeats at the N-terminus and transcription activation/repression domains at the C-terminus. Each repeat of the MYB domain is ∼52 amino acids in length and forms a helix-turn-helix (HTH) architecture (3). The three imperfect repeats (R) in *c-myb* proto-oncogene product (c-Myb), a key regulator for proliferation and differentiation of hematopoietic cells (4), are referred to as R1, R2 and R3, and other MYB proteins are classified according to their sequence similarity to these c-Myb repeats (5). Based on their sequence characteristics, MYB transcription factors are grouped into 1R, R2R3, 3R and 4R classes, which contain one to four repeats, respectively (3).

R2R3-MYB proteins only exist in terrestrial plants, but they form the largest subfamily of MYB transcription factors (2). As an example, more than 100 R2R3-MYB proteins have been identified in Arabidopsis (3). R2R3-MYB proteins play important roles in metabolism, cell fate determination, growth and development, and responses to biotic and abiotic stress (3). For example, many R2R3-MYB genes are involved in lignin or flavonoid synthesis in different species, including Arabidopsis *AtMYB32* (6), *Triticum aestivum TaMYB1D* (7), *Prunus persica PpMYB18* (8), *Chrysanthemum morifolium CmMYB1* (9) and *Pinus taeda PtMYB14* (10). In Arabidopsis, *AtMYB0* (*GLABRA1*, *GL1*) and *AtMYB23* function in trichome initiation in shoots (11) and *AtMYB66* (*WEREWOLF*, *WER*) determines root hair patterning (12). *AtMYB59* (13) and *AtMYB77* (14) modulate root growth, *AtMYB91/AS1* regulates leaf development (15), and *AtMYB21*, *AtMYB24* and *AtMYB57* control anther development (16). There are also many R2R3-MYB genes involved in stress responses: *AtMYB96* contributes to drought stress response and pathogen resistance (17,18), *AtMYB102* functions in insect defense (19), *AtMYB15* is related to cold tolerance (20), and *AtMYB62* regulates phosphate starvation responses (21).

Despite the broad range of studies on the biological functions of R2R3-MYBs in plants, the molecular basis of their recognition of target DNAs is poorly understood. Structural studies of MYB proteins have been performed mainly in animals and viruses. The structure of c-Myb was first analyzed in solution by nuclear magnetic resonance (NMR) spectroscopy, which indicated that R2 and R3 are involved in target DNA recognition (22,23). The crystal structures of mouse c-Myb (MsMyb), avian myeloblastosis virus (AMV) v-Myb and *Trichomonas vaginalis* MYB3 (TvMyb3) further revealed that the third helices of R2 and R3 recognize the target DNAs in the major groove (24,25). Although there are many more MYB members in plants than in animals, only very limited structural information is available for plant MYB proteins. The first reported plant MYB protein structure was the crystal structure of *Antirrhinum majus* RADIALIS (AmRAD) (26). Recently, the crystal structure of Arabidopsis phosphate starvation response 1 (AtPHR1) in complex with its target DNA was also determined, showing that two copies of PHR1 MYB domains bind to the major groove of DNA (27). Both AmRAD and AtPHR1 are 1R-type MYB transcription factors. However, despite being the largest group of MYB transcription factors, no structures of plant R2R3-MYB proteins have been reported so far.

To better understand how plant R2R3-MYB proteins recognize their target DNAs, we used Arabidopsis WER (AtMYB66) as a model to perform structural analysis. In Arabidopsis roots, WER is specifically expressed in non-hair cells (N-cells) and activates *GLABRA2* (*GL2*), a central regulatory gene for epidermal cell fate determination (12). Previous *in vitro* and *in vivo* studies showed that WER specifically recognizes the *cis*-element WER Binding Site (WBS) located within the *GL2* promoter and inhibits root hair formation in N-cells (28,29). Here, we report the structure of the WER–DNA complex, showing that R3 specifically recognizes the AAC element and R2 associates with the GC element in a redundant manner. The R3 residues responsible for DNA binding are conserved in both plants and animals, indicating that plant R2R3-MYBs and animal R1R2R3-MYBs recognize the AAC element in a conserved manner. In contrast, the R2 residues involved in DNA binding are variable, which probably contributes to the diversity of target DNA sequences between plants and animals. Similar to other transcription factors (30), DNA methylation at the fifth position of cytosine (5mC) in the AAC element weakened the interaction between WER and DNA. Interestingly, we found that DNA methylation at the sixth position of adenine (6mA) within the AAC element also impaired the interaction between WER and its target DNA, suggesting that 6mA modification is involved in target DNA recognition by transcription factors.

## MATERIALS AND METHODS

### Protein expression and purification

DNAs encoding 6 × His-SUMO-tagged full-length and truncated WER proteins were obtained by PCR and subcloned into the pET28a vector (Novagen, Madison, WI, USA). Constructs for WER mutants were generated by the overlapping PCR method. The primers used in plasmid construction are listed in Supplementary Table S1. A DNA encoding MsMyb (77–193) was synthesized by the GENEWIZ company (https://www.genewiz.com.cn/) and was subcloned into the pET28a vector. The resulting constructs were transferred into *Escherichia coli* BL21 (DE3) competent cells for protein expression. Protein expression was induced by adding isopropyl β-d-1-thiogalacto-pyranoside (IPTG) to a final concentration of 0.2 mM. The induced cultures were then grown at 18°C for 18 h. The cells were harvested by centrifugation and lysed by a high pressure disruptor. The homogenate was clarified by centrifugation (22 000 g) at 4°C for 1 h. The supernatant was loaded onto a Ni-NTA column. The eluted sample was dialyzed and treated with Ulp1 protease to remove the 6× His-SUMO tag. Then, the proteins were purified with a Superdex 75 16/60 preparation grade column (GE Healthcare, Milwaukee, WI, USA). The proteins were concentrated (∼30 mg/ml) and stored in a buffer of 300 mM NaCl, and 20 mM Tris–HCl, pH 8.0.

### EMSA experiment

Different quantities of WER 12–130 (from 0 to 0.8 μM) were mixed with 0.1 μM DNA in buffer (150 mM NaCl and 20 mM Tris–HCl, pH 8.0). The total volume of the reaction system was 20 μl. To investigate the effect of redox, either 2 mM DTT or 5 mM H_2_O_2_ were included in the WER 12–130 (from 0 to 0.6 μM) and DNA (0.1 μM) reaction. The samples were incubated on ice for 1 h and then analyzed on 6% native PAGE gels with 0.5× TBE buffer. The gel was stained with GelRed and imaged using a UV system.

### ITC experiments

ITC experiments were performed with a MicroCal iTC200 (GE Healthcare) by injecting WER 12–130 (400 μM), MsMyb (400 μM) or mutant protein solutions into 20 μM DNA duplex solutions. Before reaction, all protein and DNA samples were dialyzed against a buffer composed of 150 mM NaCl and 20 mM HEPES, pH 7.5. For all reactions, 24 injections (each 1.6 μl) were performed in the experiment at 25°C. Binding curves were generated by plotting the heat change of the binding reaction, and the data were fitted using one set of binding site model with Origin 7.0.

### Crystallization, data collection and structural refinement

Six truncated WER proteins (which contained amino acids 12–120, 12–130, 12–135, 9–120, 9–130 and 9–135 of WER) were expressed, purified and used in co-crystallization trials with various DNAs. The crystallization samples were prepared by mixing 15 mg/ml protein (in 150 mM NaCl, 20 mM Tris–HCl pH 8.0 buffer) with DNA at a molar ratio of 1:1.2. High quality crystals were obtained in the presence of WER 12–130 (named WER-R2R3) and dsDNA (5′-AAATTCTCCA_10_A_11_C_12_C_13_G_14_C_15_ATTTTC-3′, 5′-CGAAAATG_8*_C_9*_G_10*_G_11*_T_12*_T_13*_GGAGAATT-3′) containing an overhang. The crystals were grown by the hanging drop vapor diffusion method at 18°C, the well solution is composed of 0.1 M MES pH 6.0, 30% (v/v) PEG600, 5% (w/v) PEG1000 and 10% (v/v) glycerol.

All crystals were cryo-protected using their mother liquor supplemented with 25% glycerol and snap-frozen in liquid nitrogen. The X-ray diffraction data were collected on beamline BL17U at the Shanghai Synchrotron Radiation Facility (SSRF) at cryogenic temperatures and maintained with a cryogenic system. The complex structure was solved by the molecular replacement method using the TvMyb3 structure (PDB_ID: 3ZQC) as a search model. The structure was refined using the Refmac5 program of CCP4i (31) or the phenix.refine program of Phenix (32). The data collection and refinement statistics are summarized in Supplementary Table S2. All structure images were created with PyMOL.

### Dual luciferase assay

Arabidopsis protoplasts were isolated as previously described (33). Four-week-old rosette leaves were cut into 1 mm slices and fully immersed in protoplast enzymatic hydrolysate (0.15% (w/v) cellulase R10, 0.035% (w/v) pectolyase Y-23, 0.4 M mannitol, 20 mM KCl, 20 mM MES, 10 mM CaCl_2_, 0.02 mg/ml BSA). The protoplasts were lightly shaken for 3–4 h at room temperature while protected from light. For the dual luciferase assay, the promoter of *GL2* was amplified and inserted into the pGreenII-0800-LUC vector. The full-length CDSs of WER and its mutants fused with 3 × FLAG were amplified by nested PCR and inserted into the p1300 vector. Different combinations of the two types of plasmids were co-transferred into protoplasts transiently. After 12–16 h incubation in the dark, the protoplasts were harvested by low-speed centrifugation and quantified with a dual-luciferase assay kit (E1910.Promega). The renilla luciferase activity was used as an internal control to normalize the vector and then to normalize the luciferase activity. The relative activity values are shown as means ± SD of three independent biological replicates.

## RESULTS

### The target DNA sequences of Arabidopsis R2R3-MYB proteins

**Figure 1.**
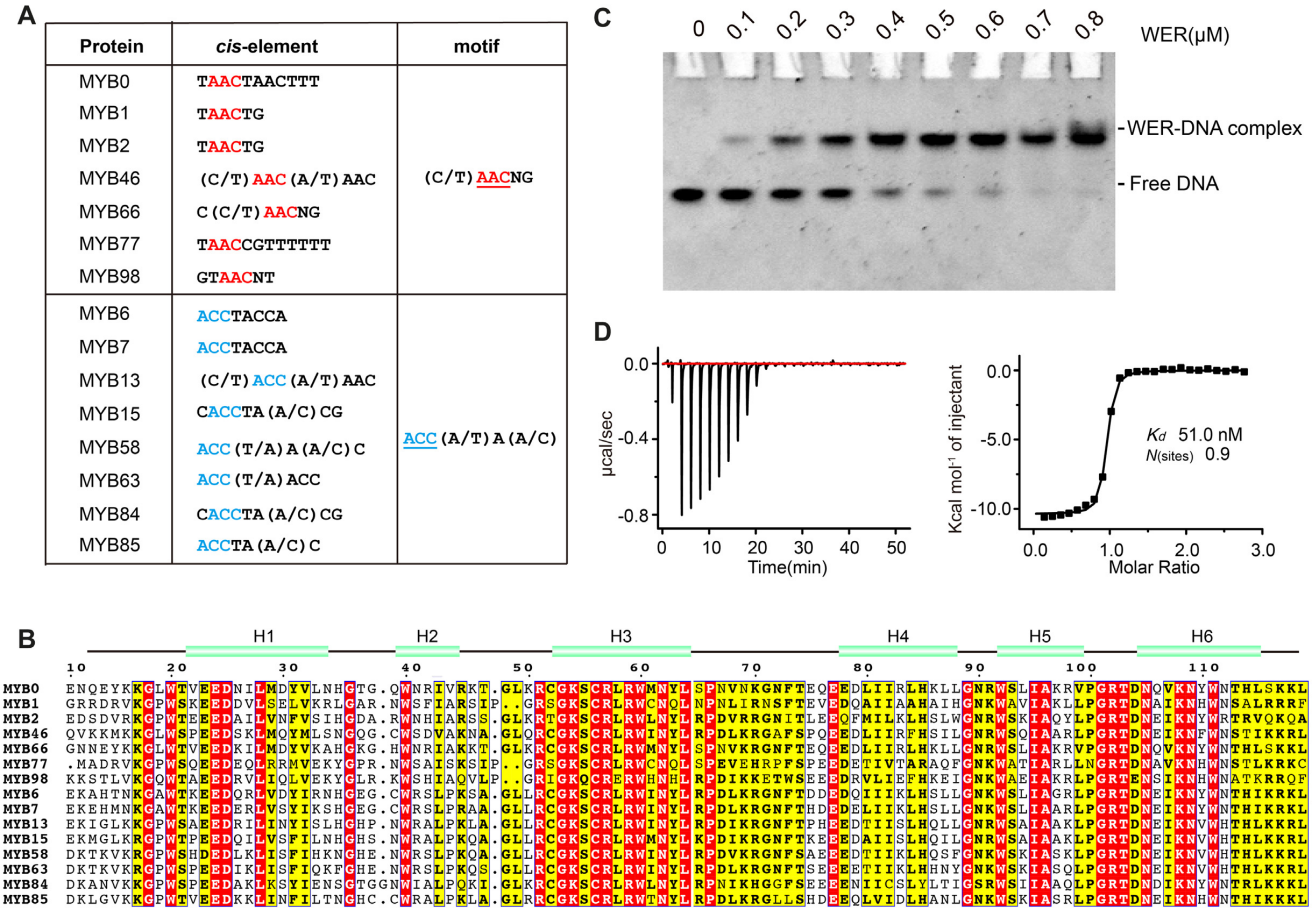
DNA motifs recognized by R2R3-MYB proteins in Arabidopsis. (**A**) Sequences of R2R3-MYB target motifs that have been verified by *in vitro* and *in vivo* assays. (**B**) Sequence alignment of typical R2R3-MYB proteins. The secondary structure of WER was predicted by Phyre^2^ program (http://www.sbg.bio.ic.ac.uk/phyre2). (**C**) EMSA (Electrophoretic Mobility Shift Assay) assay showing the interaction between WER and its target DNA (5′-AAATTCTCCAACCGCATTTTC-3′, 5′-GAAAATGCGGTTGGAGAATTT-3′). The DNA concentration was fixed at 0.1 μM and the protein concentration was increased from 0 to 0.8 μM. (**D**) ITC (Isothermal Titration Calorimetry) experiment measuring the binding affinity between wild-type WER and its target DNA (5′-AAATTCTCCAACCGCATTTTC-3′, 5′-GAAAATGCGGTTGGAGAATTT-3′).

The *cis*-elements of R2R3-MYB proteins can be grouped into two distinctive motifs: 5′-(C/T)AACNG-3′ and 5′-ACC(A/T)A(A/C)-3′ (Figure 1A), according to the previous verification *in vitro* and/or *in vivo* (34). As revealed in the structures of both MsMyb and TvMyb3 in complex with DNA, *cis*-elements are recognized by the third helices of R2 and R3 repeats. Interestingly, though their *cis*-elements are different, plant R2R3-MYB proteins share high sequence similarity in their third and sixth helices, corresponding to the third helices of R2 and R3 (Figure 1B). These observations indicate that plant R2R3-MYB proteins may be able to adopt subtle different conformation to coordinate with the corresponding *cis*-elements.

To unravel the molecular basis of target gene recognition by plant R2R3-MYB proteins, we selected WER as a model. Consistent with previous studies (28,29,35), our *in vitro* EMSA experiment (Figure 1C) confirmed that WER binds to its target DNA containing the 5′-(C/T)AACNG-3′ motif within the promoter of *GL2*. As determined by the ITC analysis (Figure 1D), the equilibrium dissociation constant (*K*_d_) value between WER and its target DNA is approximately 51 nM.

### The overall structure of the WER–DNA complex

**Figure 2.**
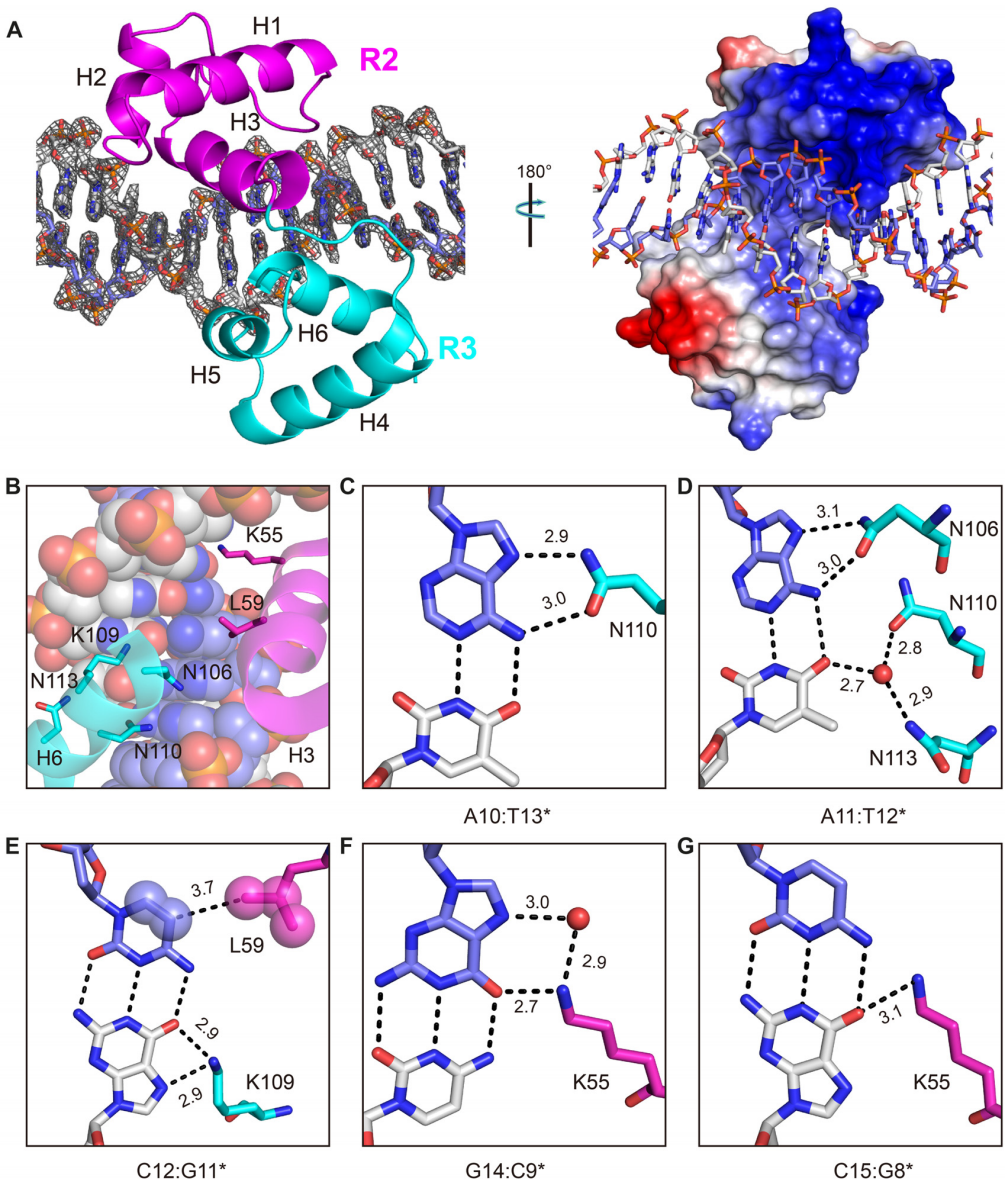
Structure of the WER–DNA complex. (**A**) The overall folding of WER–DNA complex. DNAs are shown as sticks. In the left and right panels, WER-R2R3 is shown in cartoon and as an electrostatic surface potential map, respectively. The 2F_o_-F_c_ electron density map was contoured at the 1.0 σ level. (**B**) Close-up view showing the relative orientations of the H3 and H6 helices of WER-R2R3 and DNA. The helices are shown in cartoon-and-stick form. The DNA is shown as spheres. (**C–G**) Sequence-specific recognition of A10:T13*, A11:T12*, C12:G11*, G14:C9* and C15:G8*, which correspond to the first, second, third, fifth, and sixth base pairs of the DNA, respectively. DNA base pairs and WER residues responsible for base recognition are shown as sticks. The C-atoms of R2 and R3 residues are magenta and cyan, respectively. Water molecules are shown as red spheres. The distances of the direct or water-mediated H-bond interactions are indicated by numbers.

To gain more insight into DNA recognition by WER, we purified different truncated WER proteins and performed co-crystallization trials with various DNAs. Though crystals were only obtained in the presence of WER 12–130 (named WER-R2R3) and dsDNA, they diffracted well (up to 2.15 Å). The structure was solved by the molecular replacement method and is referred to as the WER–DNA complex hereafter. The data collection and refinement statistics are summarized in Supplementary Table S2. As depicted in Figure 2A, WER-R2R3 is composed of six α-helices, H1 (V23–H36), H2 (W41–K47), H3 (G54–L65), H4 (E76–L89), H5 (W93–R99) and H6 (D105–L116). Helices H1–H3 and H4–H6 belong to the R2 and R3 repeats, respectively. R2 and R3 are connected by a 7-residue linker (_67_PNVKRGN_73_, Supplementary Figure S1A). In addition to other hydrophobic residues, each helix contains a conserved aromatic residue (Phe or Trp) forming the inner hydrophobic core of R2 (Supplementary Figure S1B) and R3 (Supplementary Figure S1C), respectively. Owing to the presence of R52, R58, and R60, the outer surface of WER-R2R3 is highly positive (Figure 2A and Supplementary Figure S2A), and the side chains of these positively charged residues form extensive H-bond interactions with DNA phosphate backbones. Several other residues such as G19, W21, S56, H40, N42, N91, W93 and S94 are also involved in DNA backbone recognition (Supplementary Figure S2B–E).

WER-R2R3 contains two Cys residues (C53 and C57), which are highly conserved in plant R2R3-MYB proteins (Figure 1B). Previous studies suggested that, via change of their redox state, these Cys residues can affect DNA binding activity of plant R2R3-MYB proteins (36). Consistent with the previous report, our EMSA analysis showed that no effect was observed on WER–DNA interaction by adding reducing agent DTT, whereas oxidizing agent H_2_O_2_ significantly blocked the interaction between WER and its target DNA (Supplementary Figure S3). In the WER–DNA complex, the CB-SG bond of C53 points toward the DNA duplex, but does not directly interact with the DNA (Supplementary Figure S4A). C57 of WER corresponds to the C130 residue of MsMyb (Supplementary Figure S4A). The CB-SG bond of C57 points toward the hydrophobic core of R2 and inserts into the pocket formed by the side chains of W21, L29, I44, L50 and R52 (Supplementary Figure S4B). Like C130 in MsMyb (24), the reduced C57 residue might also stabilize the conformation of the R2 repeat of WER.

### Sequence-specific recognition between WER and its target DNA

The WER–DNA complex structure showed that the third helices (H3 and H6) of R2 and R3 are inserted into the major groove of the DNA, producing a sequence-specific interaction with the 5′-AACCGC-3′ motif (Figure 2B–G and Supplementary Figure S2E). A10:T13* (the first base pair of the 5′-AACCGC-3′ motif) forms two H-bonds with N110 of R3; one (2.9 Å) is between the N7 atom of A10 and the ND2 atom of N110 and the other (3.0 Å) is between the N6 atom of A10 and the OD1 atom of N110 (Figure 2C). A11:T12* (the second base pair of the 5′-AACCGC-3′ motif) is also recognized by one Asn residue (N106), through two similar H-bonds between the nucleobase of A11 and the side chain of N106 (Figure 2D). To stabilize the conformation of A11:T12* base pair, the nucleobase of T12* forms two water-mediated H-bonds: one with N110 and the other with N113 (Figure 2D).

The third base pair of the 5′-AACCGC-3′ motif, C12:G11*, is recognized by K109 of R3 (Figure 2E). Instead of C12, K109 interacts with the nucleobase of the pairing G11*. The NZ atom of K109 forms H-bond interactions with both the O6 and the N7 atoms of G11*; the average distance is around 2.9 Å (Figure 2E). The side chain of L59 points toward the nucleobase of C12, and the distance between the CD2 atom of L59 and the C5 atom of C12 is 3.7 Å (Figure 2E). The nucleobases of C13:G10* do not have any direct interaction with WER. G14:C9* and C15:G8* (the fifth and sixth base pairs of the 5′-AACCGC-3′ motif) are both recognized by K55 of R2. The NZ atom of K55 forms one H-bond (2.7 Å) with the O6 atom of G14, and mediated by one water molecule, the NZ atom of K55 also interacts with the N7 atom of G14 (Figure 2F). Instead of C15, K55 forms a direct H-bond interaction with the pairing G8* of the sixth base pair (Figure 2G).

### Verification of WER–DNA interaction *in vitro* and *in vivo*

**Figure 3.**
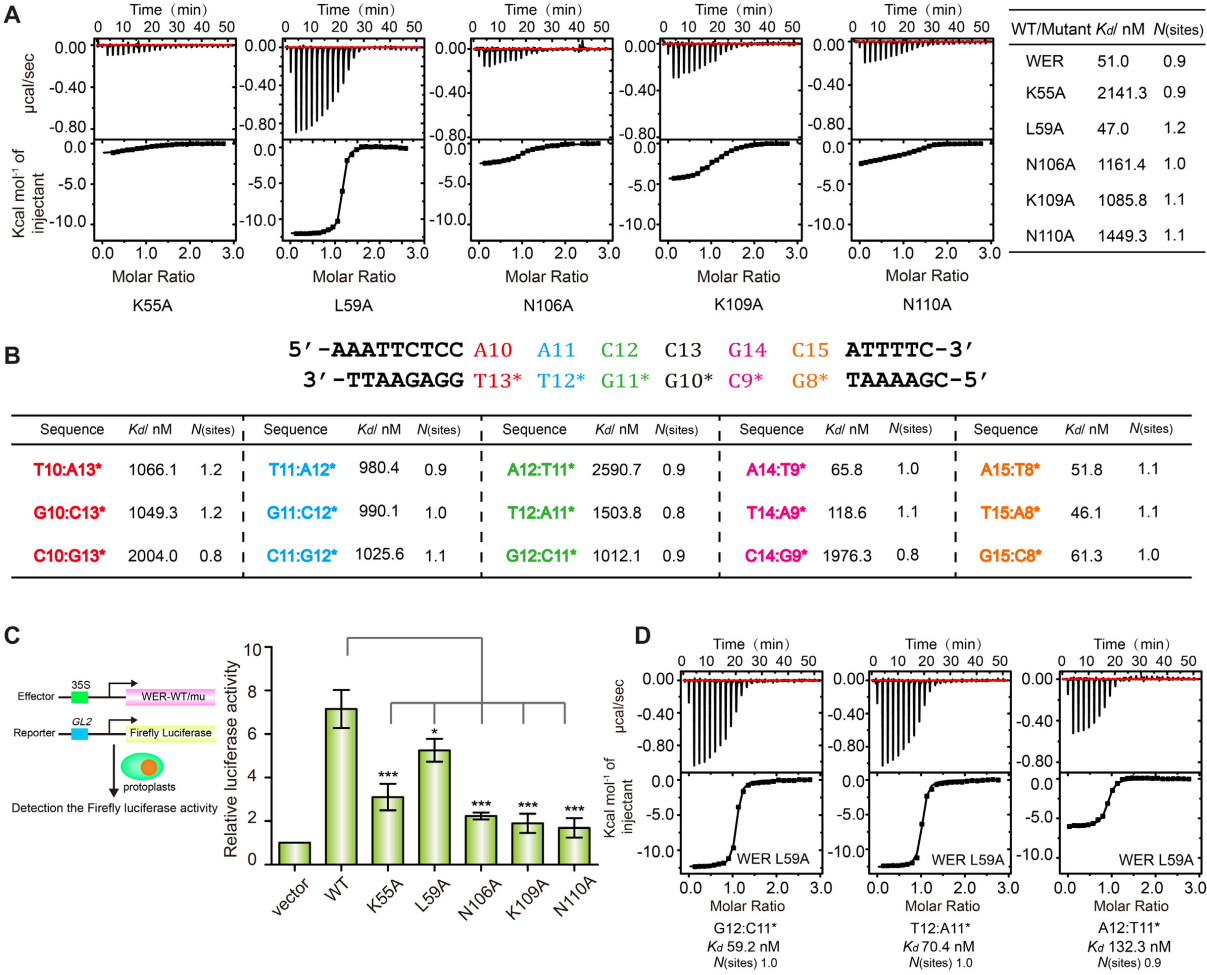
Verification of sequence-specific interactions. (**A**) ITC experiments showing the impact of mutation of WER residues involved in dsDNA (5′-AAATTCTCCAACCGCATTTTC-3′, 5′-GAAAATGCGGTTGGAGAATTT-3′) binding. (**B**) ITC experiments showing the impact of DNA core motif (5′-AACCGC-3′) mutation on WER binding. The detailed sequence of the target DNA is shown on the top of the table. For mutated DNAs, only the mutated base pairs are listed in the table for clarity. (**C**) Dual luciferase assay of the *GL2* promoter activation activity of wild-type and mutated WER proteins. Values are means ± SD of three independent biological replicates. * and *** indicate statistically significant differences between the wild type (WT) and mutant at *P* < 0.05 and *P* < 0.001, respectively. (**D**) ITC experiments showing the binding affinities of WER L59A mutant to the A12:T11*, G12:C11* and T12:A11* mutants of the 5′-AACCGC-3′ motif, respectively.

To verify the specific interactions observed in the WER–DNA complex, we constructed and purified five WER single-point mutants, in which K55, L59, N106, K109, or N110 were substituted by an Ala (A) amino acid, and performed ITC analysis (Figure 3A). Compared to that of wild-type WER, DNA binding affinities of N106A, K109A, and N10A mutants were approximately 20∼30-fold decreased, and K55A mutant caused more dramatic reduction (>40-fold). Different from other mutants, the DNA binding affinity of L59A mutant (*K*_d_: 47 nM) is comparable to that of wild-type WER (*K*_d_: 51 nM) (Figure 3A), probably because of the long distance (3.7 Å) between the CD2 atom of L59 and the C5 atom of C12 (Figure 2E).

In addition to WER, we also made systematic mutations of the DNA *cis*-element 5′-AACCGC-3′, in which the five WER-interacting base pairs (A10:T13*, A11:T12*, C12:G11*, G14:C9* and C15:G8*) were replaced by other Watson-Crick base pairs (Figure 3B and Supplementary Figure S5). As revealed by ITC analysis, any substitution within the A10:T13*, A11:T12*, or C12:G11* base pairs significantly decreased (∼20–50-fold) the binding affinity between WER and DNA. The most dramatic reduction was observed for the C10:G13* and A12:T11* mutants, *K*_d_ values of which are 2004 and 2590 nM, respectively. Replacement of G14:C9* base pair with A14:T9* or T14:A9* has no obvious impact on WER–DNA interaction, but C14:G9* mutation caused a 40-fold decrease in the DNA binding affinity of WER. Substitution of C15:G8* by all other Watson-Crick base pairs did not affect the interaction of WER–DNA (Figure 3B and Supplementary Figure S5). G14:C9* and C15:G8* are both recognized by K55 of R2. Different from many other residues, the side chain of lysine is long and flexible. We speculate that K55 of WER may undergo certain conformational changes to interact with different base pairs at either 14th or 15th position of the target DNA, such as A14:T9* and T14:A9*, and maintain the binding affinity between WER and the mutated DNAs. Taken together, these results indicated that WER specifically recognizes the 5′-AACNDN-3′ (D: A or T or G) motif.

To further verify the sequence-specific interactions identified in the structure, we used a dual-luciferase reporter assay to compare the *GL2* promoter activation activities of the wild-type and mutated WER proteins *in planta*. The *GL2* promoter containing the 5′-AACCGC-3′ motif was transferred into a luciferase reporter vector linked with the firefly luciferase reporter (Figure 3C). When the effector construct *P_35S_::WER* and the reporter construct *P_GL2_::Firefly luciferase* were co-transferred into Arabidopsis protoplasts, the activity of firefly luciferase was enhanced to 7-fold compared with the negative control (Figure 3C). Compared with wild-type WER, the *GL2* promoter activities of the WER K55A, N106A, K109A and N110A mutants (Figure 3C) were dramatically decreased, indicating that K55, N106, K109 and N110 are all essential for WER to associate with its target DNA *in planta*.

We noticed that although the *in vitro* DNA binding affinity of WER L59A mutant was comparable to that of wild-type WER (Figures 1D and 3A), the *GL2* promoter activation activity of WER L59A was weaker than that of wild-type WER *in planta* (Figure 3C). We were puzzled by this observation, so we measured the binding affinities of WER L59A to the A12:T11*, G12:C11* or T12:A11* mutants of the 5′-AACCGC-3′ DNA motif (Figure 3D). Although they were poor substrates for wild-type WER, the A12:T11*, G12:C11* and T12:A11* mutants were all good substrates to WER L59A (Figure 3D). Besides the 5′-AACCGC-3′ motif within the *GL2* promoter, we speculate that WER L59A might bind to other genes containing the sequences of 5′-AANCGC-3′, probably leading to its weak *GL2* promoter activation activity *in planta*.

### 5mC modification of the DNA blocks its interaction with R2 repeat of WER

As revealed by structural superposition (Supplementary Figure S6A), the overall folding of WER-R2R3 is similar to that of MsMyb (PDB code: 1H8A), TvMyb1 (PDB code: 2KDZ) and TvMyb3 (PDB code: 3ZQC) with root-mean-square derivations of 0.900, 1.861 and 0.673 Å, respectively. WER-R2R3 shares up to 40% sequence similarity with MsMyb, TvMyb1 and TvMyb3 (Supplementary Figure S6B). Of the six DNA-recognizing residues (K55, L59, N106, K109, N110 and N113) of WER, five are conserved in MsMyb, TvMyb1 and TvMyb3; whereas L59 of WER is substituted by a Glu (E) amino acid in all the other proteins (Supplementary Figure S6B).

**Figure 4.**
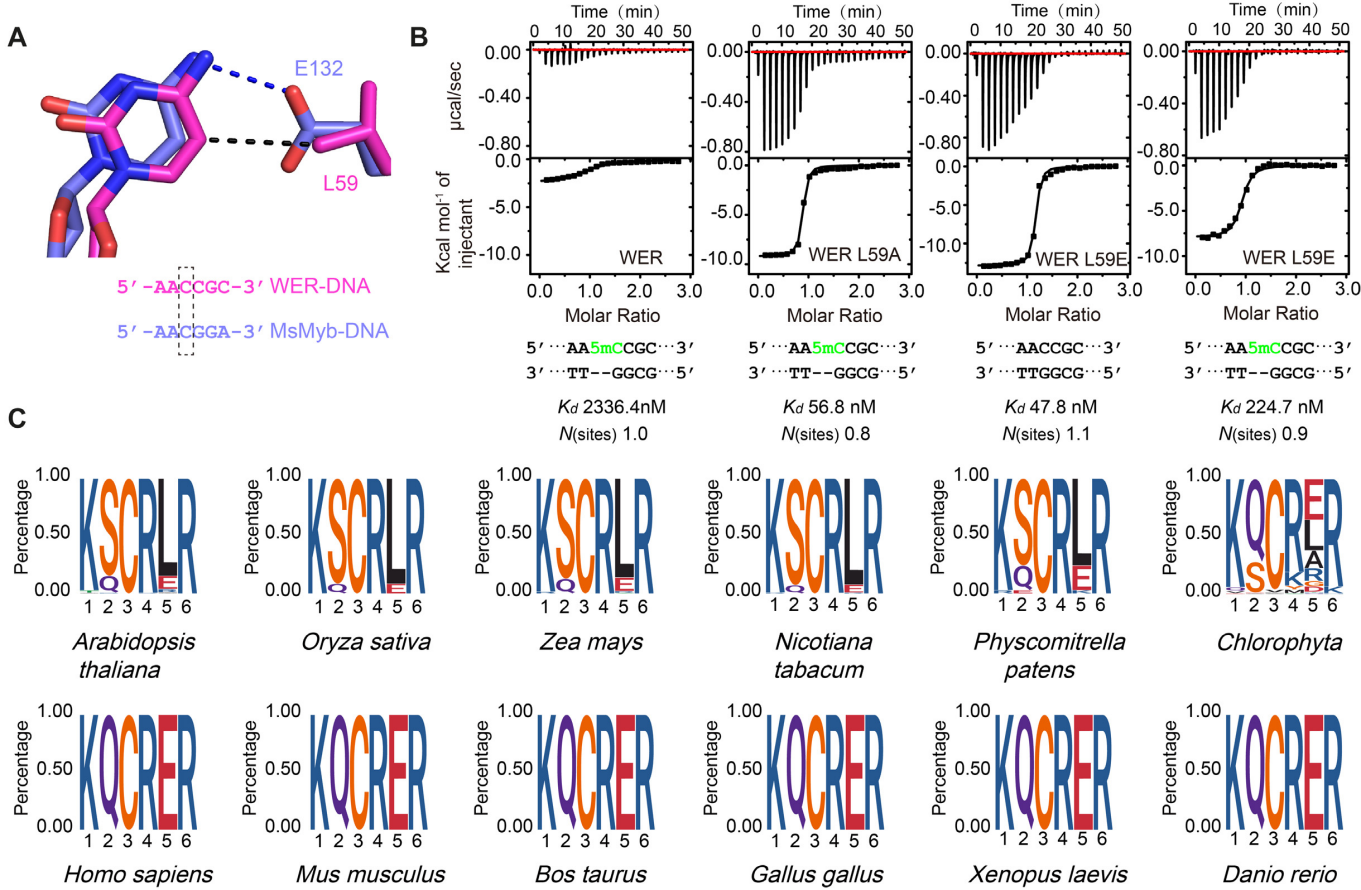
R2 repeat is incompatible with DNA 5mC modification. (**A**) The interactions between L59/E132 and the C5 atom of cytosine in WER–DNA/MsMyb–DNA (PDB code: 1H8A) complexes. (**B**) ITC analysis showing the impact of 5mC modification on DNA binding by WER and its mutants. (**C**) Consensus sequence and conservation analysis of the R2 motif involved in DNA recognition of MYB family proteins. Plant and animal MYB proteins are shown in the upper and lower panels, respectively.

The side chain of L59 points toward the nucleobase of C12 (the 12^th^ cytosine of 5′-AAATTCTCCA_10_A_11_C_12_C_13_G_14_C_15_ATTTTC-3′) in the WER–DNA complex (Figure 4A). In eukaryotes, cytosine can be methylated at the fifth position (5mC), which acts as an important epigenetic mark (37). As confirmed by complex structures, 5mC is recognized by one Ile (I) residue in many proteins (Supplementary Figure S6C). Thus, we wonder whether MYB-type transcription factors can interact with 5mC-modified DNA. To answer this question, we first performed ITC analysis using WER-R2R3 and DNA with or without methyl modification at the C5 position of the nucleotide C12. Compared to the unmodified DNA (Figure 1D), the binding affinity between the 5mC modified DNA (5′-AA5mC-3′) and WER decreased to more than 45-fold with a K_d_ value of 2336 nM (Figure 4B). Different from wild-type WER, ITC analysis showed that 5mC modification has no obvious impact on DNA binding to the WER L59A mutant (Figure 4B). Both Leu and Ile residues contain two methyl groups and are hydrophobic in nature. However, unlike Ile in which the methyl groups are attached to its CB and CG atoms, both methyl groups of Leu are attached to the CG atom, which may cause close contact with the methyl group of 5mC as remodeling in Supplementary Figure S6D, leading to decreased binding affinity between WER and 5mC-modified DNA.

Although the percentage is low, some plant R2R3-MYB members (Figure 4C upper panel) have a Glu (E) residue in the position corresponding to L59 of WER. The Leu residue is also substituted by Glu in MsMyb, TvMyb1 and TvMyb3 (Supplementary Figure S6E). In fact, evolutionary analysis revealed that this Glu residue is highly conserved in animals (Figure 4C, lower panel). In the MsMyb structure, the Glu residue (E132) forms one H-bond with the N4 atom of C20 (Figure 4A). To test whether the Glu residue can tolerate 5mC modification in the target DNA, we constructed one L→E mutant of WER (WER L59E) and performed ITC analysis. Compared with wild-type WER (Figure 1D), the WER L59E mutant showed a similar binding affinity (47.8 nM) to the unmodified DNA (Figure 4B). 5mC modification caused an ∼4-fold decrease in the DNA binding affinity for WER L59E (Figure 4B), and similar results were also observed in a previous MsMyb study (24). The L59A mutation did not affect the interaction between WER and its target DNA (Figures 1D and 3A), indicating that L59 of WER is not critical for DNA binding, which can explain why L59E mutation has no obvious impact on DNA binding by WER. Compared to Leu, the side chain of Glu is more flexible. As shown in the modeling figure (Supplementary Figure S6D), the side chain of Glu59 can easily undergo conformational change to accommodate the methyl group of 5mC, which may explain why L59E is less efficient in blocking 5mC binding. Together, our results suggested that DNA 5mC modification may block the binding of MYB transcription factors to their target DNAs in both plants and animals.

### 6mA modification of the DNA inhibits its interaction with R3 repeat of WER

**Figure 5.**
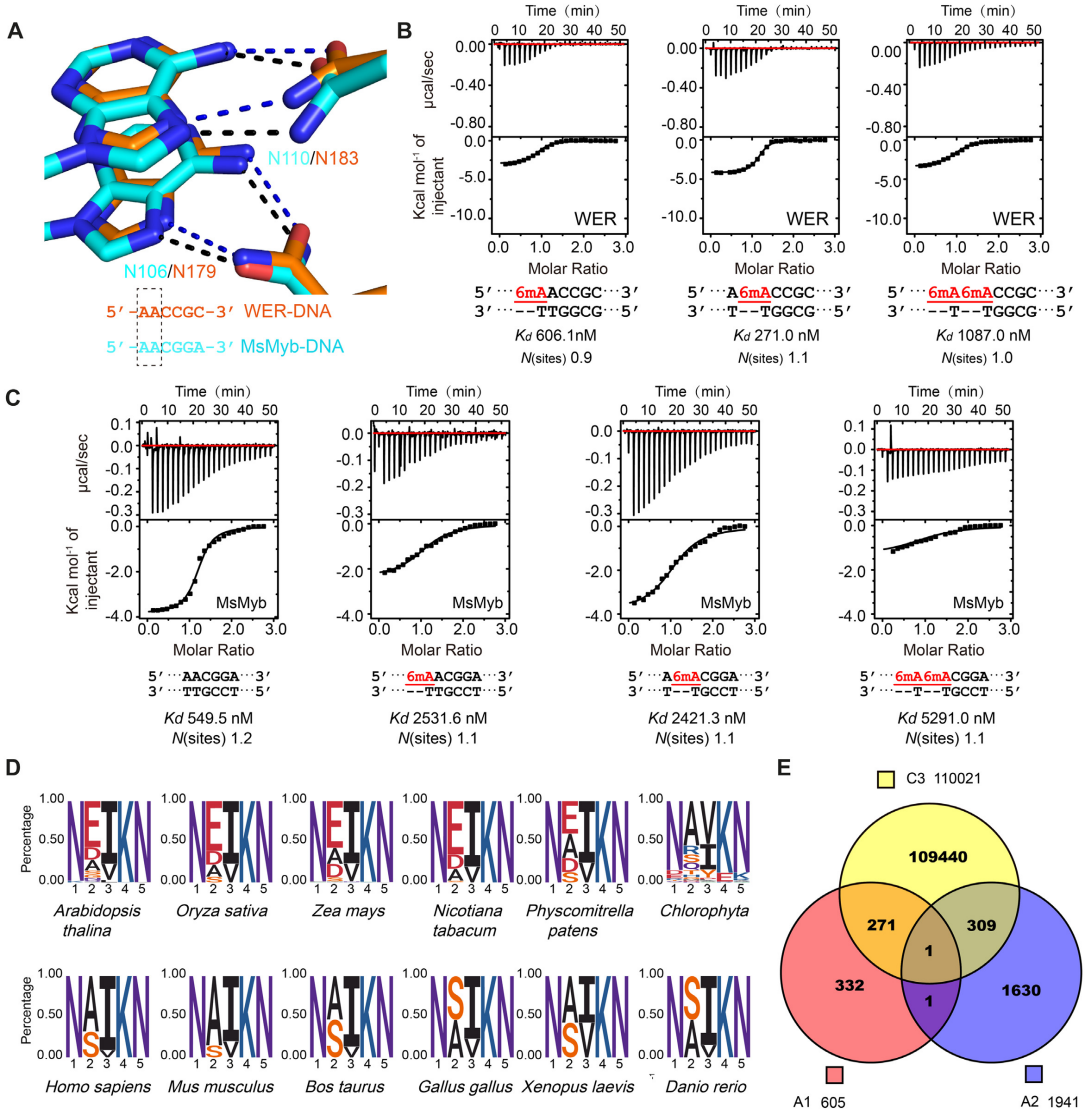
R3 repeat is incompatible with DNA 6mA modification. (**A**) The interactions between R3 Asn residues and the AA element in WER–DNA and MsMyb–DNA (PDB code: 1H8A) complexes. ITC analysis shows the impact of 6mA modification on DNA binding by (**B**) WER and (**C**) MsMyb, respectively. (**D**) Consensus sequence and conservation analysis of the R3 motif involved in DNA recognition of MYB domain proteins. Plant and animal MYB proteins are shown in the upper and lower panels, respectively. (**E**) The potential target motif AACNDN (D: A or T or G) recognized by WER can be methylated *in vivo* by searching the Arabidopsis DNA methylation databases (GSM2807190 for 5mC, GSM2157793 for 6mA).

In the structure of the WER–DNA complex, A10 and A11 (the 10^th^ and 11^th^ adenines of 5′-AAATTCTCCA_10_A_11_C_12_C_13_G_14_C_15_ATTTTC-3′) were bound by two Asn residues (N106 and N110) of WER R3 repeat. The amino groups at the 6 position in the two adenines both form direct H-bond interactions with the side chains of N106 and N110 (Figure 5A), and similar interactions were also observed in the MsMyb-DNA and TvMyb-DNA structures (Supplementary Figure S7A–C). Similar to cytosine, adenine can also be methylated. Like 5mC, methylation at the sixth position of adenine (6mA) can also function as an epigenetic mark in eukaryotes (38). To investigate whether 6mA modification can affect DNA binding by MYB proteins, we synthesized 6mA-modified DNA and performed ITC analysis (Figure 5B). Compared with the unmodified DNA, methylation of the first adenine (5′-6mAAC-3′) decreased the binding affinity between WER and its target DNA by 12-fold, and methylation of the second adenine (5′-A6mAC-3′) reduced the affinity of WER for its DNA site by 5-fold. Double methylation (5′-6mA6mAC-3′) caused more serious (>20-fold) inhibition on WER–DNA interaction. These results indicated that WER is incompatible with DNA 6mA modification.

In addition to WER, we also measured the binding affinities between MsMyb and 6mA-modified DNAs. As shown in Figure 5C, 6mA modification of either of the two adenines caused a dramatic reduction of the DNA binding affinity to MsMyb. Besides WER and MsMyb, the two adenine-interacting Asn residues are also highly conserved in other plant and animal MYB proteins (Figure 5D), suggesting that 6mA modification might be a conserved mechanism for regulating the expression of genes targeted by MYB proteins in both plants and animals.

Our WER–DNA complex structure and ITC analysis showed that WER specifically recognizes the 5′-AACNDN-3′ (D: A or T or G) motif (Figures 2 and 3). To analyze if the potential target elements of WER are methylated *in vivo*, we analyzed the AACNDN motifs genome-wide within the promoter regions by searching the Arabidopsis DNA methylation databases (GSM2807190 for 5mC, GSM2157793 for 6mA), considering that WER is a transcription factor. We found 1467251 AACNDN motifs located at gene promoter regions (0–3 kb upstream of transcription start site) of 33322 genes in Arabidopsis. Among them, 111984 AACNDN motifs are methylated including 110021 motifs with 5mC modification at the third cytosine, 605 and 1941 motifs with 6mA modification at the first and second adenine, respectively. Some motifs contain both types of methylation, for example 272 motifs with 5mC modification at the third cytosine and 6mA modification at the first adenine, 310 motifs with 5mC modification at the third cytosine and 6mA modification at the second adenine, and 1 motif with 5mC modification at the third cytosine and 6mA modifications at both the first and second adenines. Together, our results indicated that AACNDN motifs are subject to methylation *in planta* (Figure 5E).

## DISCUSSION

In this study, we determined the crystal structure of WER in complex with its target DNA, representing the first structure of R2R3-type MYB proteins. Although most of the residues involved in DNA binding are conserved, one residue (Leu versus Glu) of the R2 repeat is the major difference between plant and animal MYB proteins, which probably contributes to the variety in their target DNAs. Interestingly, we found that not only 5mC but also 6mA modifications inhibit the interactions between MYB transcription factors and their target DNAs.

The structure of the WER–DNA complex showed that the R2 and R3 repeats of WER specifically recognize the 5′-AACCGC-3′ motif. The first adenine, second adenine and the third cytosine interact with R3, and the fifth guanine and sixth cytosine are recognized by R2. However, compared to DNA containing 5′-AACCGC-3′ motif (Figure 1D), the 5′-ACCCGC-3′ containing DNA binding affinity of WER is much weaker (Figure 3B). Interestingly, though sequence alignment showed that the key residues responsible for DNA recognition are highly conserved in all R2R3-MYB proteins (Figure 1B), nearly half R2R3-MYB members recognize DNA with a 5′-ACC-3′ element (Figure 1A), suggesting that these R2R3-MYB members may undergo subtle conformational changes or possesses some unidentified features to favor 5′-ACC-3′ element.

DNA cytosine methylation (DNA 5mC) is a conserved epigenetic modification in eukaryotes. Specifically, DNA 5mC is often associated with transcription factor binding sites. For example, the transcription factor NRF1 selectively binds to unmethylated target DNA, and 5mC methylation disrupts the protein-DNA interaction *in vitro* (39). *In vivo*, NRF1 can efficiently recognize the *Asz*1 promoter to active the luciferase expression. When the *Asz*1 promoter is methylated by a CpG methyltransferase, the activation activity of NRF1 is significantly reduced (39). Consistently, deletion of three DNA methyltransferases (Dnmt3a, Dnmt3b and Dnmt1) creates a large number of novel binding sites for NRF1 in mouse ES cells (30), suggesting that DNA 5mC modifications have widely hidden NRF1 binding sites across the genome. In Arabidopsis, investigation of the global impact of DNA 5mC modification by calculating the ratio of DAP-seq or ChIP-seq binding strength at *cis*-elements showed that 72% (234 out of the total 327 analyzed members) of transcription factors are sensitive to the DNA 5mC modification and 24% (79 members) are weakly impacted, while only 4% (14 members) preferentially bind to methylated motifs (40). Our data showed that the L59 residue of the WER R2 repeat is incompatible with DNA 5mC modification in the core AAC element (Figure 4B). Indeed, DNA 5mC modifications and MYB transcription factors display opposite functions in many plant-specific processes, especially fruit ripening (41,42). Apple fruit skin anthocyanin accumulation is negatively related to the DNA methylation level but positively correlated with MYB transcription factors (43). Many R2R3-MYB proteins have been shown to promote anthocyanin accumulation (44–48), but DNA hypermethylation resulted in colorless or non-ripening fruits (49). Importantly, the L→A mutation (WER L59A) resulted in slightly stronger binding affinity than that of wild-type WER and the mutated protein was not sensitive to DNA 5mC modification (Figure 4B). This observation may provide a potential way to improve the agronomic traits of flowers or fruits.

In addition to DNA 5mC, DNA 6mA modification has been discovered in various eukaryotes, including vertebrates (frog, fish, pig, mouse and human) (50–53), plants (Arabidopsis and rice) (54–56) and fungi (57). Compared to mammals, the DNA 6mA modification level is higher in plants. In the rice genome, approximately 0.2% of adenines are modified by 6mA (56) and a similar level was also observed in Arabidopsis (54). DNA 6mA is not randomly distributed but rather located around transcription start sites in Arabidopsis (54). Additionally, increasing evidence has shown that DNA 6mA modification acts as a gene expression-associated epigenetic maker that participates in multiple cellular processes including stress responses (55,58,59), tumorigenesis (53), and neuronal development (60). However, how DNA 6mA modification affects gene expression regulation is still unclear. As revealed by the structures of both WER and MsMyb, the R3 repeat specifically recognizes the AAC element, and our ITC analysis showed that DNA 6mA modification on AAC element significantly weakens the interaction between WER/MsMyb and their target DNAs (Figure 5B and C). Searching the Arabidopsis DNA methylation databases, we find that AACNDN (D: A or T or G) motifs recognized by WER can be methylated *in planta* (Figure 5E). Taken together, our study suggests that both DNA 5mC and 6mA modifications may regulate gene expression by impairing the interaction between MYB transcription factors and their target DNAs during plant growth and development.

## DATA AVAILABILITY

Structural factors and coordinates have been deposited in the Protein Data Bank under accession code 6KKS for the WER–DNA complex.

## Supplementary Material

gkz1081_Supplemental_File
