# Supplementary material for: Structural insights into target DNA recognition by R2R3-MYB transcription factors
Source: Nucleic Acids Res. 2019 Nov 16;48(1):460–71. doi: 10.1093/nar/gkz1081 (PMC7145699; doi:10.1093/nar/gkz1081)
Supplement: gkz1081_Supplemental_File [file gkz1081_supplemental_file.docx]

**Supplemental Data**


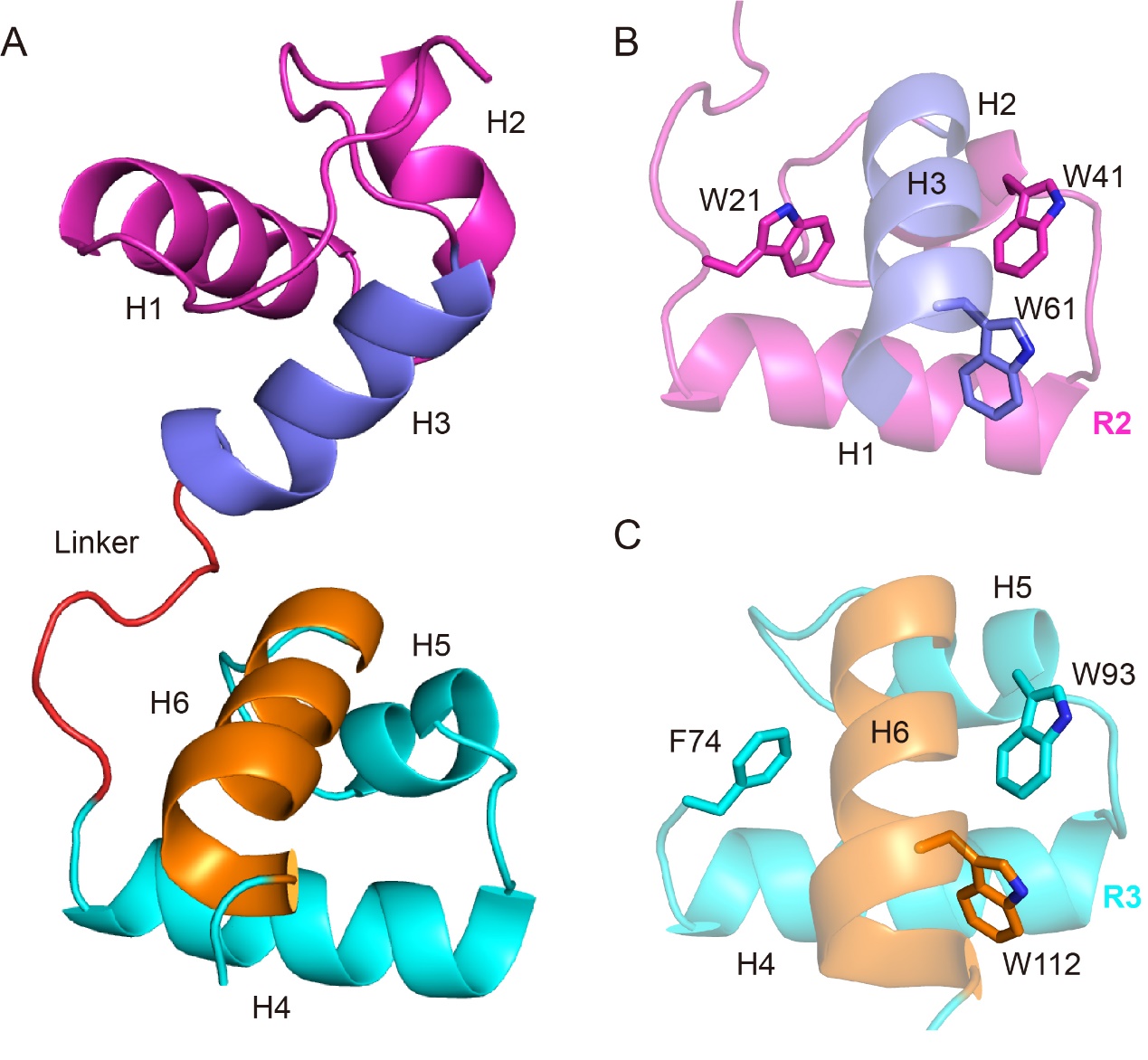


**Fig. S1.** Structure of WER-R2R3. (**A**) The overall folding of WER-R2R3. The R2 and R3 repeats are linked by a 7-residue linker, which is colored in red. The DNA-interacting helices of R2 and R3 are colored slate and orange, respectively. Conformation of the hydrophobic residues conserved in (**B**) the R2 repeat and (**C**) the R3 repeat, respectively.


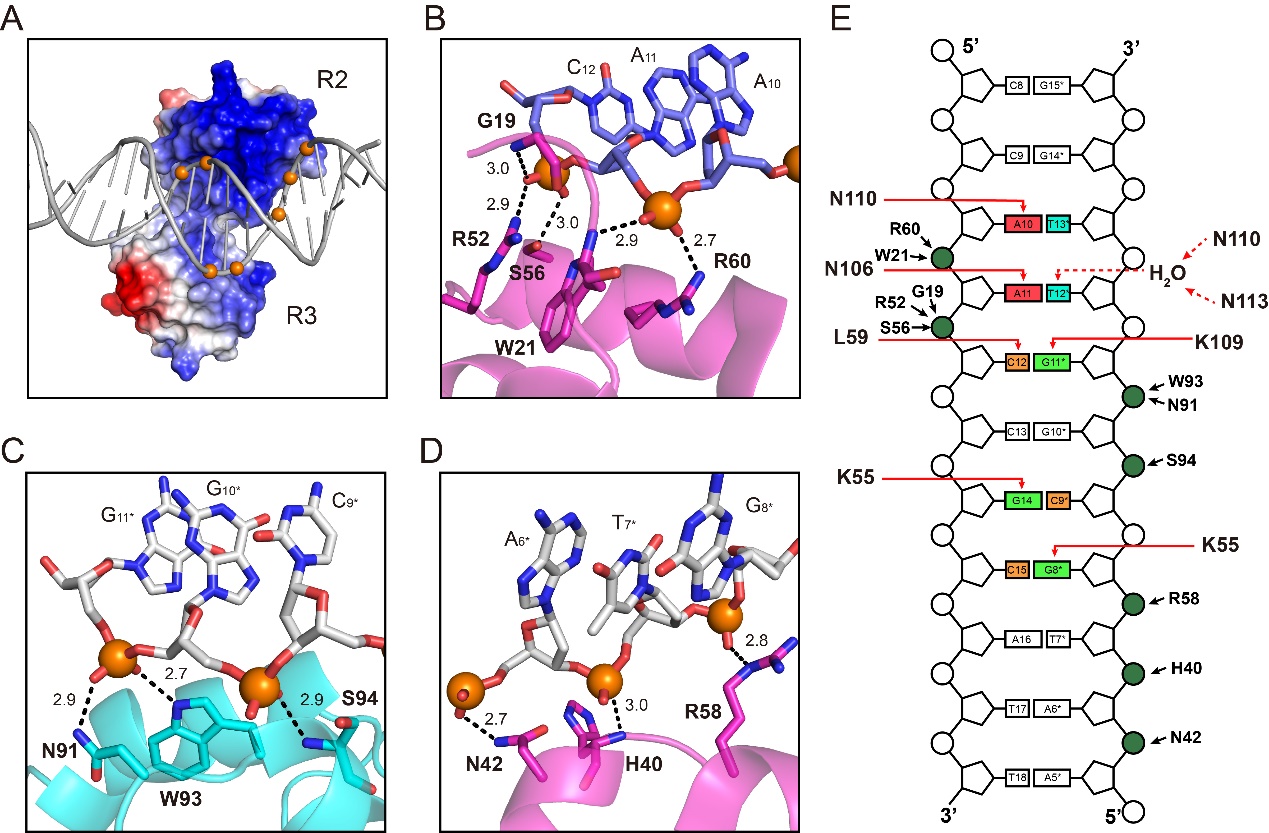


**Fig. S2.** Interaction between WER-R2R3 and the phosphate backbones of DNA. (**A**) An electrostatic potential surface map of WER-R2R3. DNA is shown in cartoon and the phosphate groups within the *cis*-element of WER are shown as orange spheres. (**B–D**) The detailed interactions between DNA phosphate backbones and the R2 and R3 residues. The phosphates in the interface are shown as orange spheres. The phosphate-interacting residues are shown as sticks. (**E**) Schematic representation of DNA recognition by WER. The base pairs involved in direct interaction are colored in red (adenine), cyan (thymine), orange (cytosine) and green (guanine).The residues G19, W21, H40, N42, R52, S56, R58, R60, N91, W93 and S94 recognize the phosphate skeleton (dark green). The red arrows depict hydrogen bonds and hydrophobic interaction, respectively.

**
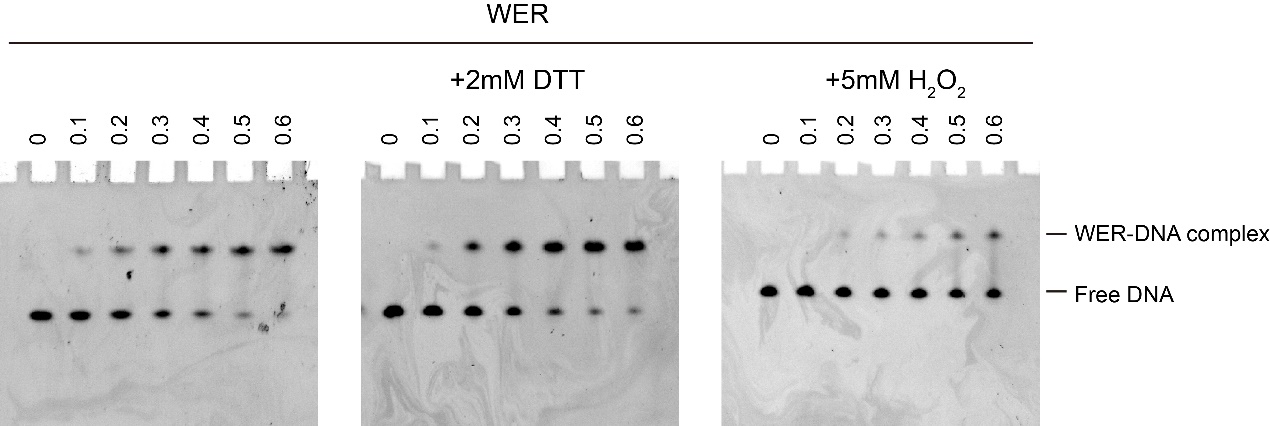
**

**Fig. S3.** The redox state is important for WER binding to its targte DNA.


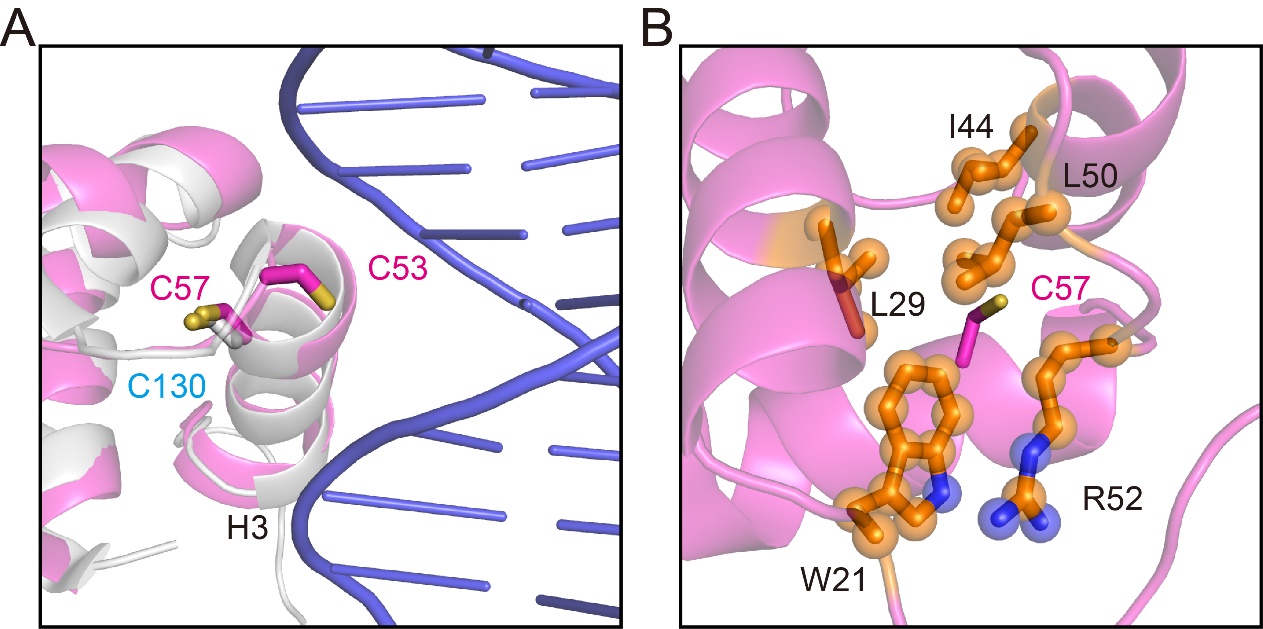


**Fig. S4.** Structural superpostion of WER–DNA and MsMyb–DNA (PDB code: 1H8A) complexes. (**A**)WER and MsMyb are magenta and white, respectively. C53 and C57 of WER are shown as sticks in atomic color (C: magenta; S: Yellow). C130 of MsMyb is also shown as sticks but its C-atoms are white. DNAs of WER–DNA complex are shown in cartoon in slate. For clarity, the DNA in MsMyb complex structure is omitted. (**B**) Sphere-and-stick view showing the binding pocket of C57.


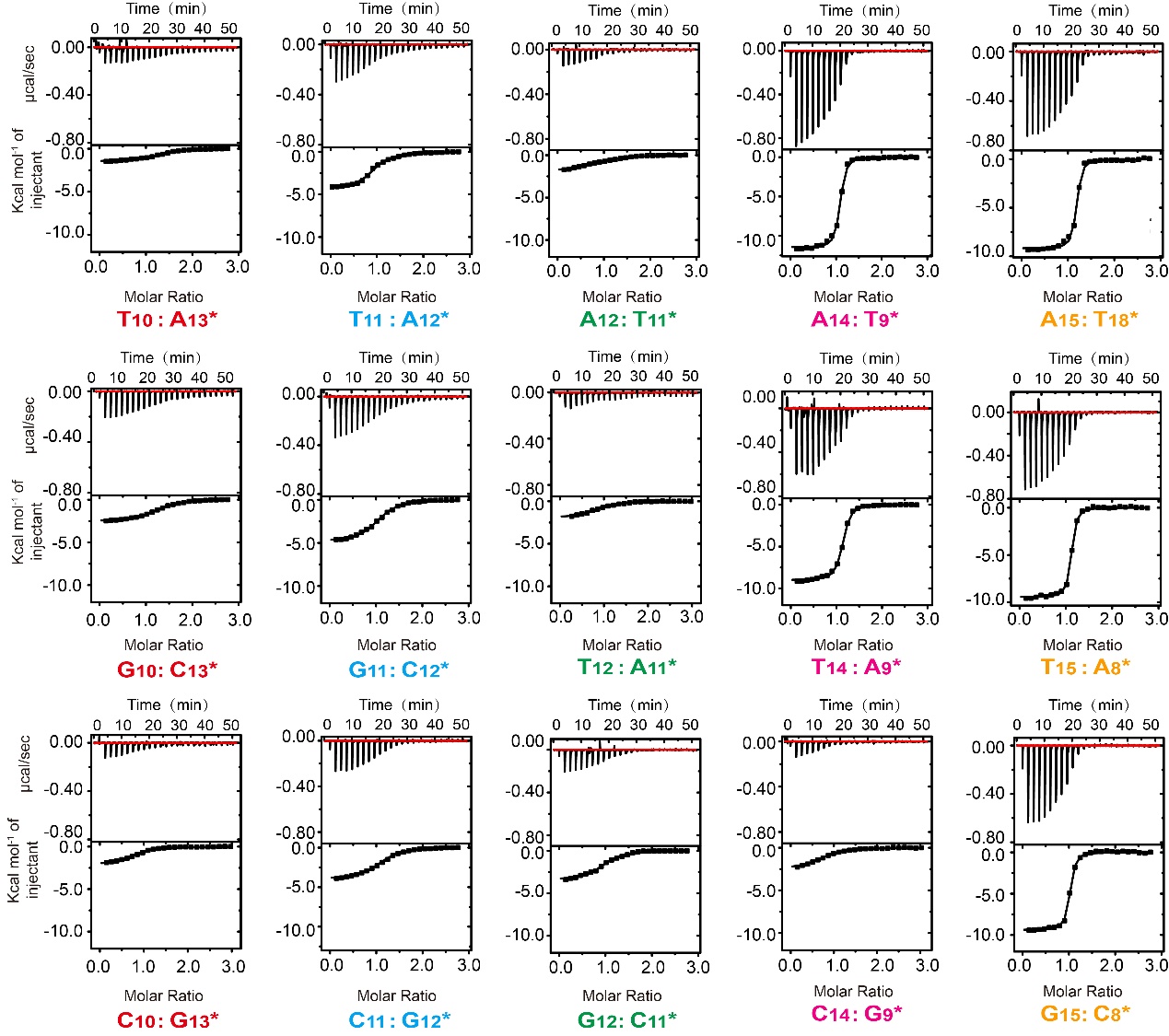


**Fig. S5.** ITC experiments showing the effects of WBS core motif (5′-AACCGC-3′) mutations on WER binding.


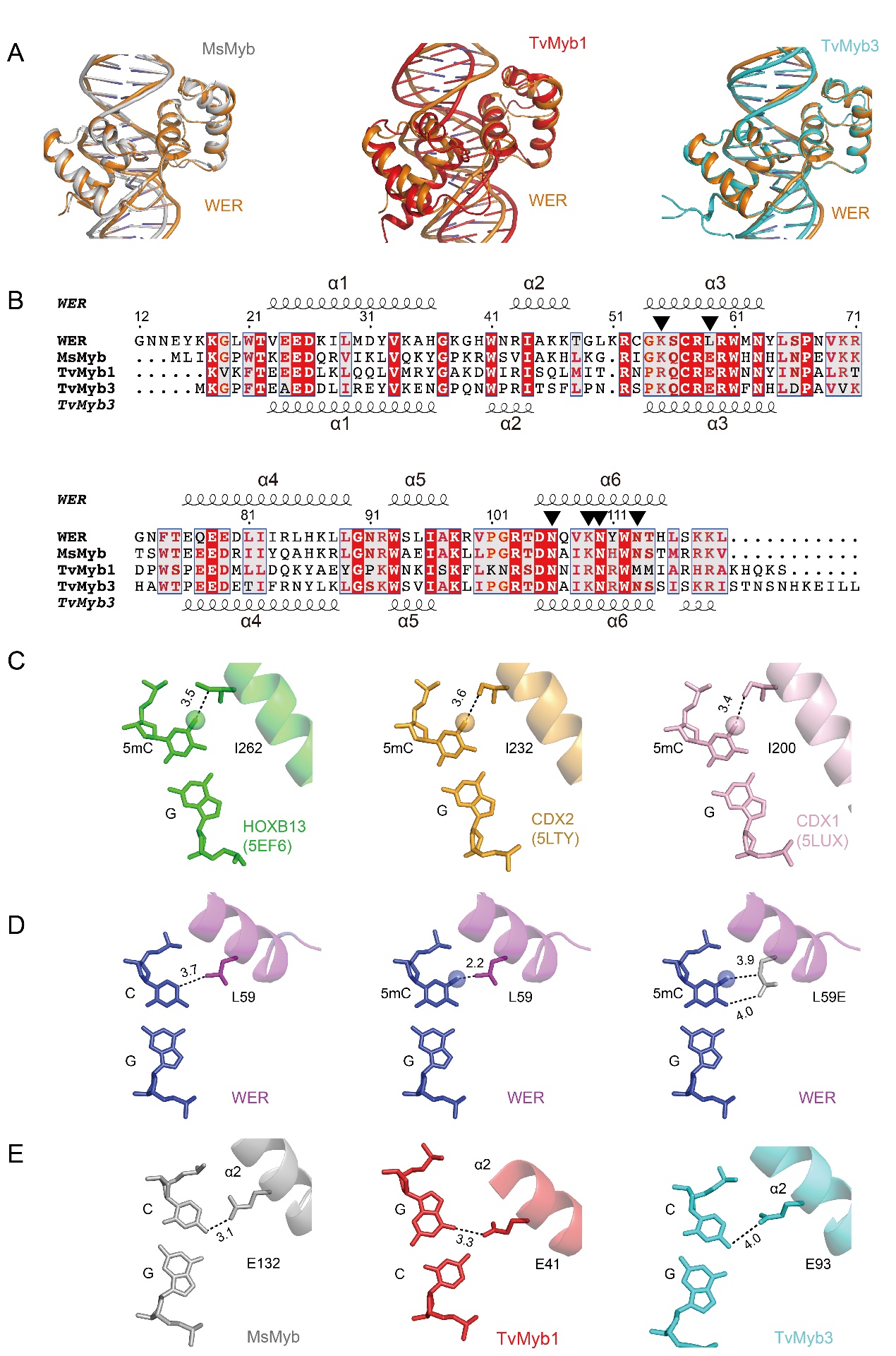


**Fig. S6.** Comparison of WER with homologous proteins. (**A**) Structural superposition of WER–DNA with MsMyb–DNA (PDB_ID: 1H8A), TvMyb1–DNA (PDB_ID: 2KDZ), and TvMyb3–DNA (PDB_ID: 3ZQC) complexes. (**B**) Sequence alignment of WER, MsMyb, TvMyb1, and TvMyb3. Residues involved in DNA sequence-specific interactions are indicated by black triangles. (**C**) Close-up view showing 5mC recognition by the Ile residue in some reported protein–DNA complex structures. (**D**) Structural modeling for the effect of 5mC modification on WER–DNA interaction. Left, interaction between L59 of WER and cytosine within the target DNA. Middle, 5mC modification can block WER–DNA interaction for the close contact of L59 with the methyl group of 5mC. Right, L59E can accommodate 5mC for the side chain of the Glu is flexible. (**E**) The detailed DNA-Glu interactions observed in MYB–DNA complex structures.


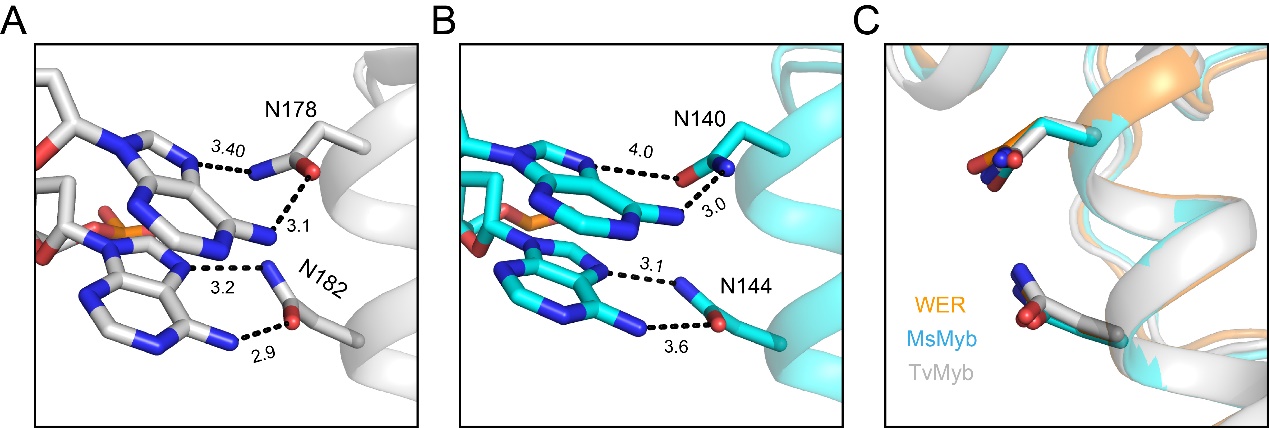


**Fig. S7.** Detailed interactions between adenine nucleobases and the conserved Asn residues of the R3 repeats in (**A**) MsMyb–DNA (PDB code: 1H8A) and (**B**) TvMyb3–DNA (PDB code: 3ZQC). (**C**) Superposition of the Asn residues conserved in the R3 repeats of MYB proteins.

Table S1. Primers used in this study.

| Name | Sequence |
| --- | --- |
| WER-12-BamHI-F | ACCAGGATCCGGAAACAATGAGTACAAGAAAGGTTTGTGGACAGT |
| WER-130-SalI-R | TACCGTCGACTCAGCTCTGTTTGGTTTTCTGATCTTTGATTCCGA |
| WER-K55A-F | ACTGGTTTAAAGAGATGTGGAGCGAGTTGTAGATTGAGGTGGA |
| WER-K55A-R | TCCACCTCAATCTACAACTCGCTCCACATCTCTTTAAACCAGT |
| WER-N106A-F | AGTGCCGGGTCGAACGGATGCTCAAGTGAAGAACTATTGGAA |
| WER-N106A-R | TTCCAATAGTTCTTCACTTGAGCATCCGTTCGACCCGGCACT |
| WER-K109A-F | GGTCGAACGGATAATCAAGTGGCGAACTATTGGAACACGCATCTTA |
| WER-K109A-R | TAAGATGCGTGTTCCAATAGTTCGCCACTTGATTATCCGTTCGACC |
| WER-N110A-F | GGTCGAACGGATAATCAAGTGAAGGCCTATTGGAACACGCATCTTA |
| WER-N110A-R | TAAGATGCGTGTTCCAATAGGCCTTCACTTGATTATCCGTTCGACC |
| WER-L59A-F | GAGATGTGGAAAGAGTTGTAGAGCGAGGTGGATGAATTATCTCAG |
| WER-L59A-R | CTGAGATAATTCATCCACCTCGCTCTACAACTCTTTCCACATCTC |
| WER-L59E-F | AGATGTGGAAAGAGTTGTAGAGAGAGGTGGATGAATTATCTCAGCCC |
| WER-L59E-R | GGGCTGAGATAATTCATCCACCTCTCTCTACAACTCTTTCCACATCT |

Table S2. Data collection and refinement statistics.

|  | | WER-DNA | | | |
| --- | --- | --- | --- | --- | --- |
| **Data collection** | | |  |  |  |
| Space group | P2_1_2_1_2_1_ | | | |  |
| Cell parameter |  | | | |  |
| a (Å)  b (Å)  c (Å) | 52.1  63.9  74.0 | | | |  |
| α (°)  β (°)  γ (°) | 90.0  90.0  90.0 | | | |  |
| Wavelength(Å) | 0.97925 | | | |  |
| Resolution (Å) | 30.0-2.15 | | | |  |
| Last shell (Å) | 2.28-2.15 | | | |  |
| Completeness (%) | 99.6(100.0) | | | |  |
| Redundancy | 14.2(14.4) | | | |  |
| I/σ(I) | 21.0(3.5) | | | |  |
| Rmerge (%)  CC(1/2) | 10.5(93.5)  0.99(0.92) | | | |  |
| **Refinement** |  | | | |  |
| Resolution (Å) | 30.0-2.15 | | | |  |
| R_work_ (%) / R_free_ (%) | 21.4/24.6 | | | |  |
| No. of atoms |  | | | |  |
| Protein | 873 | | | |  |
| DNA | 812 | | | |  |
| Water | 69 | | | |  |
| R.m.s. deviations |  | | | |  |
| Bond length (Å) | 0.006 | | | |  |
| Bond angle (°) | 1.149 | | | |  |
| Ramachandran plot (%) |  | | | |  |
| Most favored | 97.1 | | | |  |
| Additional allowed | 2.9 | | | |  |
| PDB number | 6KKS | | | |  |

*Values in parentheses are for the highest-resolution shell.
